# Evaluation of pituitary uptake incidentally identified on ^18^F-FDG PET/CT scan

**DOI:** 10.18632/oncotarget.15417

**Published:** 2017-02-16

**Authors:** Huijun Ju, Jinxin Zhou, Yu Pan, Jing LV, Yifan Zhang

**Affiliations:** ^1^ Department of Nuclear Medicine, Rui Jin Hospital, Shanghai Jiao Tong University School of Medicine, Shanghai, China

**Keywords:** Pituitary, ^18^F-FDG PET/CT, pituitary tumor, langerhans cell histiocytosis

## Abstract

The clinical significance of pituitary uptake on routine whole body ^18^F-fluorodeoxyglucose (FDG) positron emission tomography/computer tomography (PET/CT) is not completely characterized. We seek to assess the potential differential diagnosis/underlying etiology of pituitary FDG uptake incidentally identified on routine PET/CT scans. A total of 24,007 PET/CT whole body scans in recent 5 years were retrospectively reviewed. Patients with maximum standardized uptake value (SUVmax) > 4.1 in the pituitary glands were identified. Cases with a known history of pituitary disorders were excluded. Nineteen cases were identified with incidental pituitary FDG uptake which all had a final pathological diagnosis/clinical follow up. Among them, there were 9 primary pituitary tumors, with SUVmax ranging from 4.7 to 29.3 (13.6 ± 9.8); 3 metastatic malignancy with SUVmax ranging from 7.3 to 32.3 (16.0 ± 10.6); 3 Langerhans cell histiocytosis (LCH) with SUVmax ranging from 6.0 to 26.0 (15.0 ± 10.2); 1 pituitary lymphocytic hypophysitis with SUVmax of 4.7. Of note, 3 cases with SUVmax of 7.5,7.9 and 9.6 showed no relevant clinical symptoms with negative results on subsequent magnetic resonance (MR) and were counted as benign physiologic uptake. The most common differential diagnosis of incidental pituitary uptake on routine whole body PET/CT scans was primary pituitary tumors, followed by metastatic malignancy, Langerhans cell histiocytosis, and inflammatory lymphocytic hypophysitis. Of note, benign physiologic uptake without corresponding lesions could also occur in our population.

## INTRODUCTION

The pituitary gland is an important endocrine organ that releases multiple hormones, including certain tissue-targeting growth hormones and prolactin as well as pituitary hormones such as thyroid-stimulating hormone, adrenocorticotropic hormone, follicle stimulating hormone, and luteinizing hormone. Pituitary tumor is the most common disease that affects this gland, and it can be classified as either non-functional pituitary tumor or hormone-secreting tumor. In addition to pituitary lesions, other illnesses can compromise the pituitary. Pituitary gland can manifest a focal FDG uptake on ^18^F-fluorodeoxyglucose (FDG) positron emission tomographic/computed tomographic (PET/CT) imaging as an incidental finding. The clinical significance of this incidental uptake on PET/CT scans has not been completely characterized. In this study, we retrospectively examined patients who received imaging with exhibited pituitary hypermetabolism to investigate the clinical significance of this incidental FDG uptake on PET/CT scans.

## RESULTS

In this retrospective study, 32 cases out of the total 24,007 scans (0.13%) were identified to have incidental pituitary hypermetabolism. of these patients, 19 received subsequent follow-up examination and had a final diagnosis. Three patients with pituitary hypermetabolism showed no relevant clinical symptoms with negative results upon subsequent magnetic resonance (MR) examination, and were treated as benign physiologic uptake. Among the remaining 16 participants with definite diagnoses on pathological or clinical/follow-up examinations, the pituitary SUVmax ranged from 4.7 to 32.3 (15.0 ± 10.4). The differential diagnoses in descending order of prevalence were primary pituitary tumors (9/19, 47.4%), metastatic malignancy (3/19, 15.8%), Langerhans cell histiocytosis (LCH) (3/19, 15.8%), and hypophysitis (1/19, 5.3%).

The nine patients with pituitary tumor had an SUVmax ranged from 4.7 to 29.3 (13.6 ± 9.8). Five of them received surgery, including 1 prolactinomas, 1 growth hormone tumor, 2 adrenocorticotropin (ACTH)-secreting pituitary tumor, and 1 non-fonctional pituitary adenoma. All of them have received operation and confirmed by pathologic examination with immunohistochemistry. Based on tumor size, there were four cases of pituitary microadenoma (<1 cm) and five cases of pituitary macroadenoma (>1 cm), respectively.

**Figure 1:**
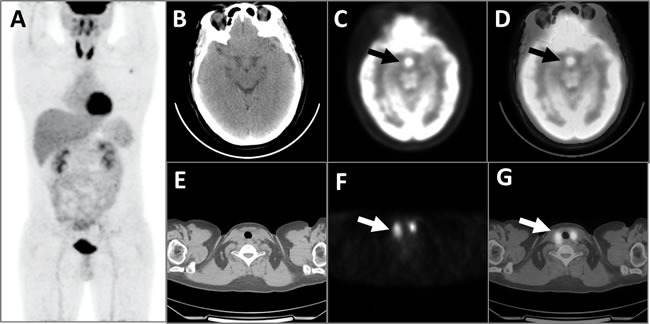
This patient with LCH had concurrent diffuse thyroid hypermetabolism The MIP image of the PET **A**. showed diffuse abnormal FDG hypermetabolism with an SUVmax of 11.3 in the residual bilateral thyroid, which was consistent with the CT scanning **E**., PET cross-section images **F**., and fusion images **G**. of the thyroid (white arrows). PET images of the brain **B**. showed significantly elevated FDG metabolic activity with an SUVmax of 12.9 (black arrows) in the pituitary, which was confirmed by the corresponding CT **C**. and fusion **D**. images.

**Figure 2:**
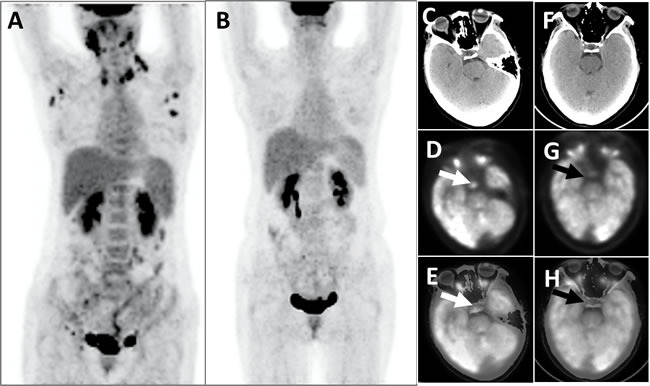
This patient with LCH developed multiple hypermetabolic lymph nodes, which disappeared after chemotherapy The PET whole-body images (MIP), taken prior to chemotherapy, showed that the patients displayed multiple abnormal FDG hypermetabolism (SUVmax = 4.4-13.2) of the lymph nodes in the bilateral submandibular areas, neck, armpits, abdomen, retroperitoneum, pelvic cavity, and bilateral inguinal areas **A**.; Post-chemotherapy PET whole-body images did not identify aberrant FDG hypermetabolic foci **B**. Head PET **D**. and **G**., CT **C**. and **F**. and PET/CT fusion topographic images (E and H) revealed that the pituitary exhibited apparent FDG hypermetabolism (white arrows, SUVmax = 6.0) before chemotherapy and that the metabolic activity was clearly decreased after chemotherapy (black arrows, SUVmax = 3.6).

The three patients with LCH had an SUVmax ranged from 6.0 to 26.0 (15.0 ± 10.2). In addition to pituitary hypermetabolism, one patient had concurrent diffuse thyroid hypermetabolism, with a postoperative residual thyroid SUVmax of 11.3 (Figure 1). The second patient was found multiple hypermetabolic lymph nodes in the bilateral submandibular regions, neck, armpits, abdomen, retroperitoneum, pelvis, and bilateral groins, with SUVmax ranged from 2.0 to 13.2 (Figure 2). In addition to multiple hypermetabolic lymph nodes in or around the neck, hilar and mediastinum, retroperitoneum, iliac vessels, and groin (SUVmax = 15.6˜18.9), the third patient also had concurrent thyroid hypermetabolism (SUVmax = 7.7), spleen enlargement and hypermetabolism (SUVmax = 8.0), local bone destruction and hypermetabolism (SUVmax = 8.7) around the left iliac wing and left pubis [1]. All the three patients had a definitive diagnosis established via a pathological examination based on other tissues’ biopsy other than pituitary.

The one case of pituitary inflammation (SUVmax = 4.7) was confirmed to be lymphocytic hypophysitis via a pituitary biopsy.

Finally, three cases of malignant tumor combined with systemic metastases and pituitary hypermetabolism were detected. The primary tumors were one endometrial cancer, one lung cancer, and one scalp melanoma, respectively, with associated pituitary SUVmax ranged from 7.3 to 32.3 (mean = 16.0 ± 10.6). There was corresponding abnormal uptake in these primary malignant sites.

## DISCUSSION

The pituitary gland is located in the sellaturcica at the base of skull, and it is covered by the dura mater. Under physiological conditions, its volume is small and commonly it is thought to manifest a background level in ^18^F-FDG PET/CT imaging [2]. In the current study, we showed that the incidental pituitary FDG uptake was 0.13% in our patient cohorts. The most common disease was primary pituitary tumor (9/19, 47.4%), including micro and macro adenomas, followed by metastatic malignancy (3/19, 15.8%), Langerhans cell histiocytosis (LCH) (3/19, 15.8%), and inflammatory hypophysitis (1/19, 5.3%). Of note, 3 patients with focal uptake showed no pathological lesions on MR nor clinical symptoms, and its uptake was within physiologic range, which is different from what had been generally known [2]. Our findings are not completely consistent with the results from Hyun SH et al who reported that the prevalence of pituitary hypermetabolism was approximately 0.8%,of which 40.8% were pathological types [3]. The difference likely reflects the differential patient population.

Pituitary adenoma accounts for 15˜20% of all cases of brain tumor and often manifests as pituitary dysfunction with an injured nervous system (particularly featuring vision damage) or the encasement of the sellaturcica and sphenoid [4]. Tumors with endocrine functions mostly manifest via abnormal blood serum hormone levels. However, cases in which the pituitary tumors neither invade the neighboring tissues nor secrete hormones typically do not perceive an anomaly.

In this study, we found 9 cases of pituitary tumor out of a total of 19 cases with incidental pituitary FDG uptake, of which three patients had pituitary microadenoma. ^18^F-FDG PET/CT imaging revealed clear pituitary hypermetabolism, with an SUVmax of 13.4 ± 9.5. Three patients in this cohort had secretory adenoma, including one case of growth hormone tumor, one case of prolactinoma combined with a growth hormone-secreting tumor, and one case of Cushing's disease. The remaining 6 patients did not exhibit relevant clinical symptoms nor abnormal laboratory findings.

Although there were several reports of pituitary metastasis of lung cancer and breast cancer, but malignant pituitary metastases are rare, and diabetes insipidus is the most seen manifestation [5].

**Figure 3:**
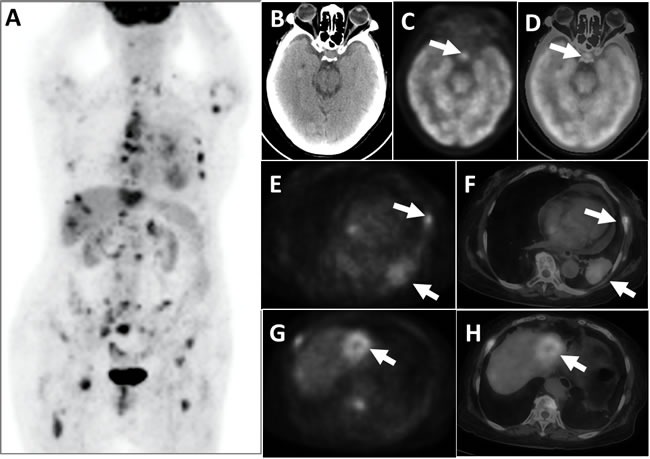
This patient suffered from lung cancer, combined with multiple systemic metastases, had pituitary hypermetabolism PET whole-body images (MIP) appeared multiple hypermetabolic **A**. PET cross-sectional imaging and CT fusion images revealed space-occupying lesions on the left lower lobe of the lung combined with adjacent rib bone destruction and hypermetabolism **E**. and **F**., white arrows); a space-occupying hypermetabolism was visible on the left liver lobe **G**. and **H**., and liver metastasis was suspected (white arrows). Brain PET **C**., CT **B**., and the fusion of cross-sectional tomographic images **D**. revealed that the pituitary exhibited FDG hypermetabolism (white arrows), with an SUVmax of 7.3.

One out of the 3 patients in this study who showed systemic metastases of malignant tumors (Figure 3) developed diabetes insipidus. It is difficult to differentiate pituitary metastases from pituitary tumors based on an ^18^F-FDG PET/CT-derived SUVmax. Reliable diagnosis might be generated by taking the local infiltration in the sella area, the patient's clinical history, and other imaging data into account. All of the 3 patients had corresponding abnormalities of their malignancies on the whole body PET/CT scan, which facilitated the pituitary metastasis diagnosis.

In addition to pituitary tumor, some other diseases, such as LCH, can also increase ^18^F-FDG uptake in the pituitary PET/CT imaging and company with similar symptoms. LCH refers to a set of diseases with unknown causes but all featured with Langerhans cell proliferation. In addition to Langerhans cell proliferation, patients can also display an increased abundance of eosinophils, monocytes, polynuclearhistiocytes, neutrophils, and lymphocytes as well as fibrosis in its advanced disease stage.

The prevalence of LCH is five cases per 1,000,000 people, and it is more common seen in children [6]. The disease most commonly involves the bones (in 75% of cases), and every bone can be affected [7–8]. The next most common LCH types can involve the skin, lung, liver, spleen, and lymph nodes. The hypothalamus, pituitary, or thyroid could also be affected but with less incidence [9]. LCH in the central nervous system (CNS-LCH) is rare and primarily found on the sella, where the most common lesions occur on the infundibulum but can also appear on chiasm, the hypothalamus, or meninges around suprasellar cistern. The featured manifestation of the disease is central diabetes insipidus [10].

In this study, all 3 patients with LCH developed diabetes insipidus as the major manifestation and exhibited pituitary nodules upon brain magnetic resonance imaging (MRI). ^18^F-FDG PET/CT imaging showed that they all displayed pituitary hypermetabolism, with an average SUVmax of 15.0 ± 10.2. Other signs such as hypermetabolism in the thyroid and lymph nodes as well as bone destruction combined with hypermetabolism were also identified. Two patients were found multiple hypermetabolic lymph nodes in their necks, mediastinum, abdomens, pelvic cavities, and groins as well as lesions in their spleens and bones. The diagnosis of LCH was established via biopsies of their necks and inguinal lymph nodes. These three patients with LCH received chemotherapy regimens of cladribine, Ara-c + VP, and COP for 3 months and experienced apparent remission of their symptoms and signs.

Clinically, the underlying etiology of diabetes insipidus is often not easily identified. Therefore, ^18^F-FDG PET/CT imaging might be useful in showing the uptake of the pituitary and other organs such as thyroid and neck lymph nodes where aspiration biopsies might be chosen to help the diagnosis. Lymphocytic pituitary inflammation is a rare autoimmune illness that is primarily characterized by pituitary lymphocytic infiltration. Its clinical signs include intracranial space occupying lesions (accompanied by headache and visual impairment) and the inadequate secretion of pituitary hormones. Its imaging manifestation is pituitary enlargement, thus it is easily confused with other pituitary diseases such as pituitary tumor or LCH.

Pituitary inflammation generally shows a positive prognosis after standard hormone treatment; therefore, invasive operations are generally not recommended. Currently, achieving reliable diagnosis by integrating various examinations without resorting to unnecessary pituitary biopsy is critical. PET/CT based diagnoses of lymphatic pituitary inflammation are seldom adopted. There was one report in which PET/CT imaging was performed on a patient with melanoma undergoing ipilimumab treatment, and the PET/CT result showed pituitary hypermetabolism. Combined with blood tests and diagnostic therapeutics, the patient was eventually diagnosed as lymphatic pituitary inflammation [11].

## MATERIALS AND METHODS

### Patients

Between November 2010 and February 2016, 24,007 patients received whole-body ^18^F-FDG PET/CT imaging in our Hospital and were retrospectively assessed for abnormal pituitary metabolism based on the maximum standardized uptake value (SUVmax) criterion of >4.1 proposed by Hyun et al [3]. The study was approved by the institutional ethical committee.

### PET/CT imaging and data processing

All the cases were performed on a Discovery VCT 16 PET/CT scanner (GE, USA) with an ^18^F-FDG (Shanghai Kexin Pharmaceutical Co., Ltd.) radiochemical purity of >95%. Each patient fasted for at least 6h with blood sugar < 7.8 mmol/L before administration of ^18^F-FDG (0.12˜0.15 mCi/kg, 4.44-5.55MBq/Kg) intracenously. CT images were first acquired using the following settings: tube voltage = 120˜140 kV, tube current = 180˜300 mA, section thickness = 3.75 mm, and pitch = 1.375. The range of scanning extended from the parietal region to the middle femur. After acquiring CT images, 3D PET images were acquired in the same area with 5-9 beds. Each cerebral bed was scanned for 5 min, and each body bed was scanned for 2-3 min. After data acquisition, the PET images were subject to attenuation correction before image reconstruction and fusion using computer software.

### Image analyses

Two experienced nuclear medicine physicians evaluated all the PET/CT scans. The most commonly adopted indicator in clinical practice, semi-quantitative SUVmax, was used as the major parameter to assess pituitary metabolic function. SUVmax was generated such that a circular region of interest (ROI; 1 cm in diameter) was plotted on the cross-section before calculation was performed using the following equation: SUVmax = tissue radioactivity concentration/(administration dose/weight).

### Statistical analysis

All data were processed using Microsoft Excel ver.14.0.7166.5000 (Microsoft Corporation. Redmond, WA, USA), and all values were expressed as mean ± standard deviation (SD).

## CONCLUSIONS

It is important to understand the differential diagnosis/etiology of incidental pituitary uptake on routine whole body FDG PET/CT, although its incidence is low. Our results showed that the most common type is the primary pituitary tumors, followed by metastatic malignancy, and LCH. Inflammatory hypophysitis is rare but still in the differential. Further differential diagnosis could be made by additional clinical, laboratory and imaging findings of each disease. Of note, different from what had been generally accepted, pituitary gland can also exhibit physiologic focal uptake without any underlying lesions and clinical manifestations.

## Abbreviations

FDG: fluorodeoxyglucose
PET/CT: Positron emission tomography/computer tomography
SUVmax: maximum standardized uptake value
LCH: Langerhans cell histiocytosis
CNS: central nervous system
MR: magnetic resonance
ACTH: adrenocorticotropin
ROI: region of interest
SD: standard deviation
